# HOUSING CONTEXT AND FUNCTIONAL HEALTH AMONG LOWER INCOME OLDER ADULTS

**DOI:** 10.1093/geroni/igz038.3509

**Published:** 2019-11-08

**Authors:** Noah J Webster

**Affiliations:** University of Michigan, Ann Arbor, Michigan, United States

## Abstract

The characteristics of where older adults live have strong links with disability. Although lower income older adults experience disability at higher rates, less is known about the link between housing characteristics and functional health in this group. A within group comparison among this population is needed to understand how aspects of this vulnerable subgroup’s housing context are associated with health outcomes. The present study examines the association between housing and functional health among a U.S. nationally representative sample of independent living (i.e., not living in nursing homes or assisted living facilities) lower income adults age 65+. Using data from round one of the National Health and Aging Trends Study, a sub-sample of N=2,865 lower income (<$15,000 in the past year) older adults was selected for analysis. Regression analyses indicate that lower income older adults living in multiunit buildings reported better functional health compared to those in other housing contexts (e.g., free-standing homes). This link also significantly varied by age and gender. Living in multiunit housing was associated with better functional health among those age 90+, not associated among those age 80-90, and was negatively associated among those age 65-79. In terms of gender, the link between multiunit housing and better functional health was only significant among women. Findings highlight variation in health across lower income older adults’ housing contexts. Potential explanatory mechanisms (e.g., social isolation) will be discussed. Such information can inform senior housing policy regarding best approaches to providing housing for older adults that optimizes and promotes independent living.

